# Safety and efficacy of peptide receptor radionuclide therapy with ^177^Lu-DOTA-EB-TATE in patients with metastatic neuroendocrine tumors

**DOI:** 10.7150/thno.77219

**Published:** 2022-09-06

**Authors:** Yuanyuan Jiang, Qingxing Liu, Guochang Wang, Huimin Sui, Rongxi Wang, Jiarou Wang, Jingjing Zhang, Zhaohui Zhu, Xiaoyuan Chen

**Affiliations:** 1 Department of Nuclear Medicine, Beijing Key Laboratory of Molecular Targeted Diagnosis and Therapy in Nuclear Medicine, Peking Union Medical College Hospital, Chinese Academy of Medical Sciences and Peking Union Medical College, Beijing 100730, China; 2 State Key Laboratory of Complex Severe and Rare Diseases, Beijing 100730, China; 3 Nanfang PET Center, Nanfang Hospital, Southern Medical University, Guangzhou, 510515, China.; 4 Department of Diagnostic Radiology, Yong Loo Lin School of Medicine and Faculty of Engineering, National University of Singapore, Singapore, 119074, Singapore; 5 Clinical Imaging Research Centre, Centre for Translational Medicine, Yong Loo Lin School of Medicine, National University of Singapore, Singapore 117599, Singapore; 6 Nanomedicine Translational Research Program, NUS Center for Nanomedicine, Yong Loo Lin School of Medicine, National University of Singapore, Singapore 117597, Singapore; 7 Departments of Chemical and Biomolecular Engineering, and Biomedical Engineering, College of Design and Engineering, National University of Singapore, Singapore 117597, Singapore

**Keywords:** peptide receptor radionuclide therapy (PRRT), ^177^Lu-DOTA-EB-TATE, ^177^Lu-DOTA-TATE, neuroendocrine tumor

## Abstract

**Rationale:** This study aimed to assess the safety, efficacy, and survival of ^177^Lu-DOTA-EB-TATE in patients with metastatic neuroendocrine tumors (NETs)**.**

**Methods:** Thirty patients with metastatic NETs were prospectively enrolled and treated with ^177^Lu-DOTA-EB-TATE (3 intended cycles at 8 to 12-week intervals, 3.7 GBq/cycle). Treatment-related adverse events were graded according to the National Cancer Institute Common Terminology Criteria for Adverse Events (CTCAE), version 5.0. The treatment response was graded according to RECIST 1.1 and PERCIST 1.0 criteria. Kaplan-Meier analysis was performed to calculate progression-free survival (PFS) and overall survival (OS).

**Results:** Patients tolerated therapy well without acute adverse effects. During peptide receptor radionuclide therapy (PRRT), no grade 4 toxicity was observed in any of the patients; grade 3 hematotoxicity was recorded in 4 patients, including grade 3 thrombocytopenia in 4 patients (13.3%) and grade-3 anemia in 1 patient (3.3%); grade 3 hepatotoxicity was recorded in 1 (3.3%) patient, and no grade 2/3/4 nephrotoxicity was observed. On long-term follow-up, none of the patients developed grade 4 hematotoxicity or nephrotoxicity of any grade, reversible grade 3 hematotoxicity (thrombocytopenia) occurred in 1 patient. There was no incidence of leukemia or myelodysplastic syndrome for the duration of follow-up. Of 27 patients with RECIST-measurable disease, partial response and stable disease were seen in 9 and 14 patients, respectively, resulting in a response rate of 33.3% and disease control rate of 85.2%. Of 29 patients evaluable for response on ^68^Ga-DOTATATE PET/CT, 14 had partial response and 11 had stable disease, with a response rate of 48.3% and disease control rate of 86.2%. The follow-up period ranged from 5 to 57 months after the first ^177^Lu-DOTA-EB-TATE PRRT with a median follow-up of 46 months. The median PFS was 36 months, and the median OS was not reached. Ki-67 index of greater than 10% was associated with poorer PFS (*P* = 0.012).

**Conclusions:** Our results suggest that PRRT with approximately 3.7 GBq ^177^Lu-DOTA-EB-TATE has acceptable toxicity profile and is effective in treating metastatic NET with high disease control rate. In addition, ^177^Lu-DOTA-EB-TATE achieved a favorable survival outcome with encouraging PFS.

## Introduction

Neuroendocrine tumors (NETs) represent a heterogenous group of neoplasms that arise from neuroendocrine cells throughout the body. The incidence rate of these tumors has increased substantially in recent years 1, 2. The choice of appropriate treatment in patients with unresectable metastatic NET is limited, and over 50% of NET patients are at an advanced stage at the time of diagnosis 3.

The majority of NETs overexpress somatostatin receptors (SSTRs), predominantly subtype 2 (SSTR2), which provides the basis for the use of SSTR2-targeted peptide receptor radionuclide therapy (PRRT). PRRT is a form of radiolabeled somatostatin analogue therapy, delivering radionuclides directly to NET tumor cells with high expression of SSTRs. At present, ^177^Lu-DOTATATE is the most frequently used radiopharmaceutical in patients with advanced NETs. Increasing evidence has confirmed the safety and efficacy of PRRT with ^177^Lu-DOTATATE for patients with advanced, progressive, and SSTR-positive NETs 4-6. In the prospective Phase 3 NETTER-1 trial, ^177^Lu-DOTATATE plus 30 mg long-acting octreotide demonstrated remarkedly longer progression-free survival and overall survival in midgut NET patients compared to 60 mg long-acting octreotide, which led to the approval of ^177^Lu-DOTATATE (Lutathera) by the U.S. Food and Drug Administration (FDA) and the European Medicines Agency (EMA) 7.

A potential disadvantage of ^177^Lu-DOTATATE is its rapid elimination from the blood stream, leading to insufficient tumor uptake and retention 8. To improve the efficacy of PRRT, Evans blue modified octreotate was synthesized and conjugated to DOTA chelator (DOTA-EB-TATE). Evans blue reversibly binds to serum albumin 9, 10, thus extending the effective plasma half-life of ^177^Lu-DOTA-TATE. In preclinical and first-in-human dosimetry studies,^ 177^Lu-DOTA-EB-TATE demonstrated much higher tumor dose compared with ^177^Lu-DOTATATE 11, 12. Our prior dose escalation study showed that ^177^Lu-DOTA-EB-TATE with doses of 3.7 GBq/cycle achieved acceptable safety and satisfactory therapeutic efficacy after one-cycle treatment, and the more recent study of ^177^Lu-DOTA-EB-TATE with escalating doses in multiple cycles further supported this result 13, 14. In the present study, we aimed to further investigate the safety, efficacy, and long-term outcome of ^177^Lu-DOTA-EB-TATE with planned dose of 3.7 GBq/cycle in patients with metastatic NETs.

## Materials and Methods

### Patients

This clinical trial was approved by the Institutional Review Board of the Peking Union Medical College Hospital, Chinese Academy of Medical Sciences & Peking Union Medical College; written informed consent was obtained from all patients. The study was registered at ClinicalTrials.gov (NCT03478358). From August 2017 to November 2021, 30 patients (17 men and 13 women, age range, 15‒69) with metastatic NETs were prospectively enrolled in this trial to evaluate the safety and efficacy of repeated cycles of ^177^Lu-DOTA-EB-TATE. The inclusion criteria for PRRT were as follows: (1) histologically confirmed NETs with high SSTR2 expression determined by ^68^Ga-DOTATATE PET/CT imaging (modified Krenning score 3 = lesion uptake > liver; or 4 = lesion uptake ≥ spleen^15^), performed within 2 weeks before therapy; (2) white blood cell (WBC) ≥ 3 × 10^9^/L; (3) platelets (PLT) ≥ 60 × 109/L; (4) hemoglobin (Hb) ≥ 90 g/L; (5) serum creatinine level < 150µmol/L; (6) patient's readiness to provide clinical information and follow-up. Exclusion criteria were as follows: (1) patients with no or low SSTR-expression on ^68^Ga-DOTATATE PET/CT (Krenning score less than 3); (2) pregnancy; (3) breast-feeding patients; (4) patients with known previous malignancies.

### Treatment regimen

DOTA-EB-TATE was labeled with ^177^Lu using a previously published method 16. The radioisotope ^177^Lu, in the form of ^177^LuCl_3_ was purchased from LuMark®, IDB, Holland. Briefly, DOTA-EB-TATE (100 µg dissolved in 20 µL of absolute ethanol) was added to 200 µL 0.5 M NaOAc (pH 5.6), and then, the required amount of radioactivity of ^177^LuCl_3_ was added. The mixture was heated at 100 °C for 30 min and then purified by a preconditioned C18 light SEP-PAK cartridge and passed through a 0.22 µm aseptic filtration membrane directly into a sterile vial. The quality control was performed with analytical thin-layer chromatography (Bioscan, USA). CH_3_OH:NH_4_OAc (v/v 1:1) was used as the developing solution. The radiolabeling yield was greater than 90% and the radiochemical purity of ^177^Lu-DOTA-EB-TATE was more than 95%. The administration of radiopharmaceutical was performed in the ward. Pre-set dose of radiopharmaceutical was slowly administered intravenously over 30 min. In order to protect the kidneys, the mixture of arginine and 5% glucose solution (25 g/L, 1000 mL) was administered at least 30 min before radiopharmaceutical administration and lasted for 4 h. The planned administered dose per cycle was 3.7 GBq. The treatment was planned for up to 3 cycles, and cycles were repeated at intervals of 8 to 12 weeks.

### Safety evaluation

All patients were clinically monitored during the administration of radiopharmaceutical and for 3 days thereafter as inpatients. Laboratory examinations including hematological parameters, renal function tests, and liver function tests were performed before, 2 weeks, and 4 weeks after each cycle during the course of PRRT, as well as during follow-up (at 4‒6 months intervals after completion of PRRT). Short- and long-term adverse events were graded in accordance with the National Cancer Institute Common Terminology Criteria for Adverse Events version 5.0 (CTCAE 5.0). The rates of adverse events at baseline, 2 weeks, and 4 weeks after each cycle of therapy, and during follow-up were recorded.

### Response evaluation

The treatment response assessment was performed 2-3 months after each cycle of PRRT. RECIST 1.1 criteria was applied to evaluate morphological response following PRRT with contrast-enhanced CT or MR. The molecular response was evaluated according to PERCIST 1.0 criteria 17 with ^68^Ga-DOTATATE PET/CT. Partial response was defined as ≥ 30% reduction in tracer uptake, progressive disease was defined as ≥ 30% increase in tracer uptake or appearance of new lesions; and stable disease as neither partial response nor progressive disease on ^68^Ga-DOTATATE PET/CT.

### Progression-free survival and overall survival

Progression-free survival (PFS) was defined as the start of the first ^177^Lu-DOTA-EB-TATE PRRT cycle to date of radiographically confirmed progression by RECIST 1.1 or PERCIST 1.0 criteria. Overall survival (OS) was defined as the start of the first PRRT to death or last follow-up.

### Statistical analysis

All statistical analyses were carried out using SPSS Statistics for Windows version 26.0 (IBM Corp, Armonk, NY, USA). Continuous variables were expressed as mean ± standard deviations; non-continuous variables were expressed as counts and proportions. Differences between paired samples before and after treatment were compared using nonparametric testing or Student's test. Univariate Cox proportional hazards regression model was used to perform the survival analysis. Kaplan-Meier curves were used to depict the survival. All statistical tests were 2-tailed, and a *P* value less than 0.05 was considered statistically significant.

## Result

### Patients

The details of patients' baseline clinical characteristics are listed in Table 1 (Supplemental Table 1). In total, 30 patients who met the eligibility criteria were enrolled (17 men, 13 women). Of 30 patients, 18 patients (60%) had pancreatic NET, and the remaining 12 patients included rectal NET (n = 5), small intestinal NET (n = 4), paraganglioma (n = 2), and unknown primary site with metastases (n = 1). World Health Organization grades of tumor differentiation included grade 1 in 4 patients, grade 2 in 24 patients, and grade 3 in 2 patients. Approximately 3.7 GBq per cycle (average: 3.84 ± 0.70) of ^177^Lu-DOTA-EB-TATE was administered. Of 30 patients, 17 did not complete 3 cycles of ^177^Lu-DOTA-EB-TATE. Among those patients, 3 patients discontinued treatment because of hematotoxicity (grade 3 thrombocytopenia). Two patients withdrew voluntarily. Eight patients were delayed due to the COVID-19 lockdown. One patient discontinued treatment because of late enrollment time. Two patients discontinued treatment due to disease progression. One patient died due to pulmonary infection. The median cumulative administered activity was 8.97 GBq (range, 3.19‒14.32 GBq) over a median of 2 cycles (range, 1‒3). Treatment cycles and cumulative administered activity are summarized in Table 2.

### Safety evaluation

Radiopharmaceutical administration was well tolerated without any serious acute adverse events. During the course of PRRT, no life-threatening grade 4 toxicity was observed in any of the patients. Grade 3 hematotoxicity was recorded in 4 patients (4/30, 13.3%), including grade 3 thrombocytopenia in 4 patients (13.3%), and grade 3 anemia in 1 patient (3.3%), who presented with grade 3 anemia already at baseline. There was a significant reduction in WBC counts (before therapy: 5.7 ± 2.0; after therapy: 4.3 ± 1.4 × 10^9^/L, *P* = 0.034), platelet counts (before therapy: 245.4 ± 99; after therapy: 150.6 ± 97.8 × 10^9^/L, *P* < 0.001), and hemoglobin (before therapy: 136.4 ± 13.7; after therapy: 120.2 ± 17.4 g/L, *P* = 0.017). Transient grade 3 hepatotoxicity (elevation of alanine aminotransferase, aspartate aminotransferase, and bilirubin) after the first cycle occurred in 1 patient, which spontaneously recovered to normal before the second cycle. No CTC-2/3/4 renal toxicity was observed in any of the patients. There was no statistically significant change observed in serum alanine aminotransferase (30.7 ± 17.4 *vs.* 36.1 ± 24.5, *P* = 0.094), aspartate aminotransferase (34.1* ±* 13.6* vs.* 34.4* ±* 17.1,* P =* 0.178)*,* alkaline phosphatase (136.1 ± 105.3 *vs.* 170.7 ± 165.5, *P* = 0.098), and creatinine level (70.6 ± 19.5 *vs.* 63.5 ± 9.0, *P* = 0.087) before and after treatment. The short-term toxicities were summarized in Table 3.

Long-term toxicity data were available for 29 patients. Long-term hematological event (thrombocytopenia, leukopenia, or anemia), occurring 7, 12, 7, 13, 23, and 21 months after the termination of PRRT, was documented in 6 patients. None of the patients developed grade 4 hematotoxicity. One patient (3.4%) had grade 3 hematotoxicity (thrombocytopenia). Five patients had grade 2 hematotoxicity, including leukopenia in 3 (10.3%), and anemia in 2 (6.9%). Three patients had grade 1 hematotoxicity, including leukopenia in 1 (3.4%), thrombocytopenia in 1 (3.4%), and anemia in 1 (3.4%).

There was no incidence of myelodysplastic syndrome or leukemia during the follow-up period. There was also no nephrotoxicity of any grade observed on long-term follow-up. Regarding hepatoxicity, only one patient had transient grade 1 toxicity (elevation of alanine aminotransferase). The long-term toxicities were summarized in Table 4.

### Efficacy of ^177^Lu-DOTA-EB-TATE

**Figure 1:**
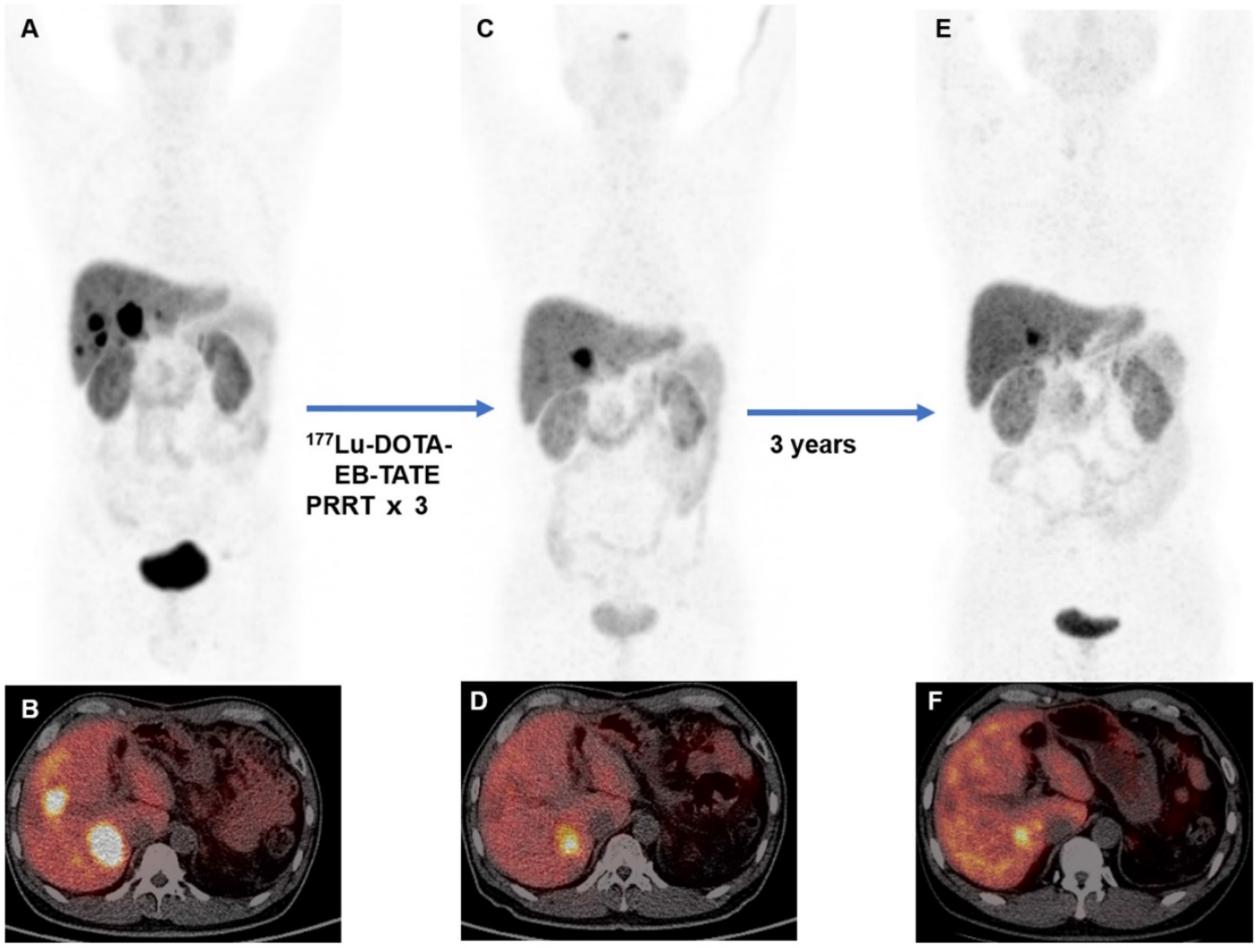
A 45-year-old man with metastatic pancreatic neuroendocrine tumor (G1; Ki-67, 1%). The baseline ^68^Ga-DOTATATE PET/CT (**A**, MIP image; **B**, fused PET/CT) showed somatostatin receptor expression in liver metastases. The patient was treated with 3 cycles of ^177^Lu-DOTA-EB-TATE with cumulative administered activity of 12.4 GBq. Two months post-therapy ^68^Ga-DOTATATE PET/CT (**C**, MIP image; **D**, fused PET/CT) showed partial response. ^68^Ga-DOTATATE PET/CT at 3-year follow-up showed stable disease (**E**, MIP image; **F**, fused PET/CT) with progression-free survival of 43 months from the first cycle of ^177^Lu-DOTA-EB-TATE PRRT.

Of the 30 patients enrolled, tumor response assessment based on RECIST criteria was available for 27 patients after ^177^Lu-DOTA-EB-TATE PRRT. Nine patients had partial response and 14 patients had stable disease, resulting in a response rate of 33.3% and a disease control rate of 85.2%. Four of those 27 patients had progressive disease. Tumor response assessment based on ^68^Ga-DOTATATE PET/CT was available for 29 patients after ^177^Lu-DOTA-EB-TATE PRRT. The partial response was seen in 14 patients, stable disease in 11 patients, and progressive disease in 4 patients, with a response rate of 48.3% and disease control rate of 86.2%. According to the primary site, pancreatic NET and non-pancreatic NET showed no statistically significant difference in treatment response referring to RECIST (response rate: 33.3% vs. 33.3%, *P* = 1.000; disease control rate: 88.9% vs. 77.8%, *P* = 0.582) and PERCIST criteria (response rate: 58.8% vs. 33.3%, *P* = 0.264; disease control rate: 94.1% vs. 75%, *P* = 0.279). Representative cases of ^177^Lu-DOTA-EB-TATE PRRT efficacy on ^68^Ga-DOTATATE PET/CT are shown in Figure 1 and Supplemental Figure 1.

**Figure 2:**
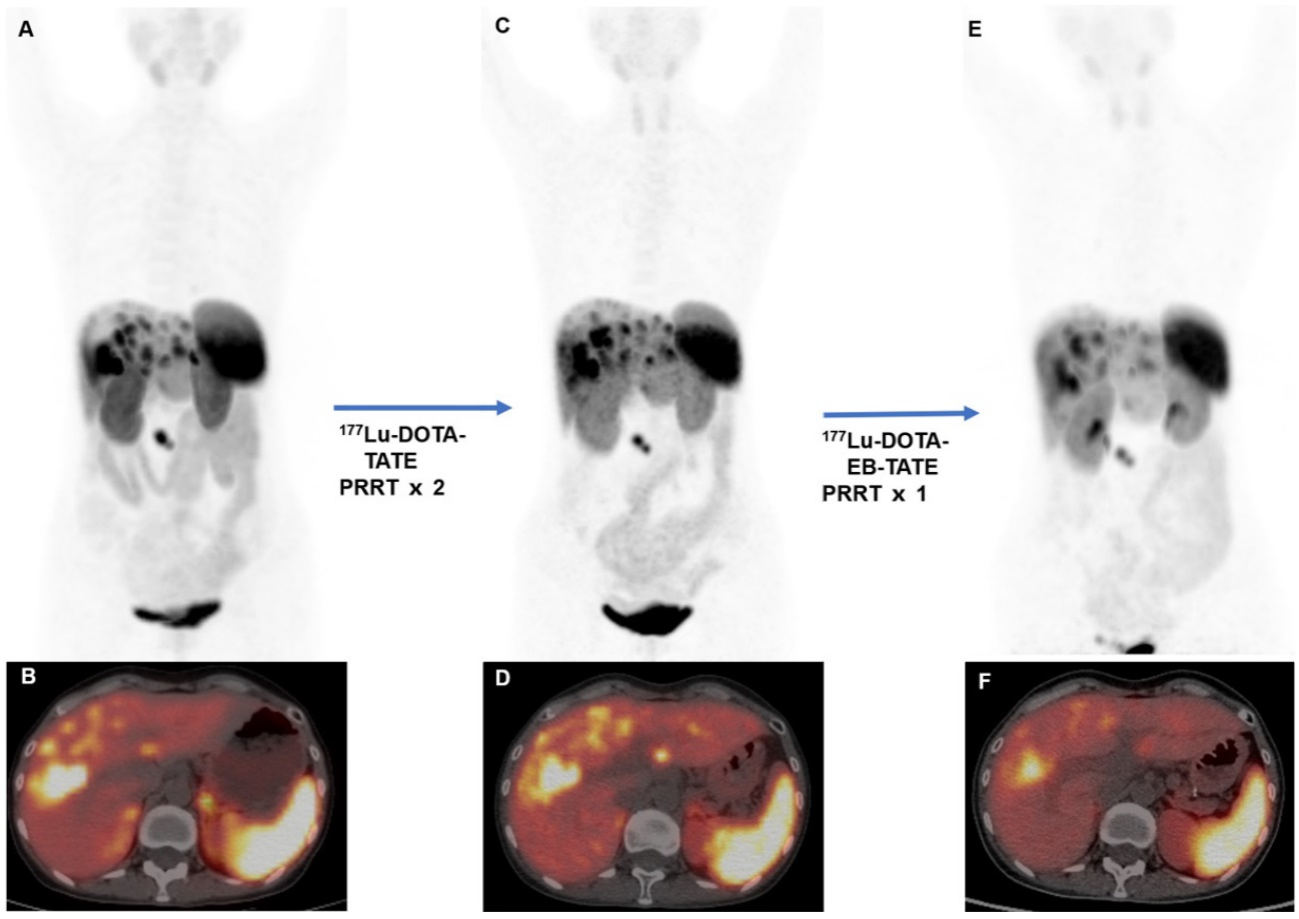
A 51-year-old woman with metastatic pancreatic neuroendocrine tumor (G2; Ki-67, 5%). The patient was previously treated with 2 cycles of ^177^Lu-DOTATATE PRRT (cumulative activity, 14.8 GBq). The baseline ^68^Ga-DOTATATE PET/CT (**A**, MIP image; **B**, fused PET/CT) before ^177^Lu-DOTATATE demonstrated somatostatin receptor avid multiple metastases. Follow-up ^68^Ga-DOTATATE PET/CT (**C**, MIP image;** D**, fused PET/CT) performed 2 months after 2 cycles of ^177^Lu-DOTATATE showed stable disease. The patient was then admitted for ^177^Lu-DOTA-EB-TATE. Follow-up ^68^Ga-DOTATATE PET/CT (**E**, MIP image;** F** fused PET/CT) performed 2 months after ^177^Lu-DOTA-EB-TATE showed partial response (administered activity, 3.85 GBq). On follow-up, the disease remained stable with progression-free survival of 15 months.

Four patients received PRRT before (PRRT re-treatment). Three patients were previously treated with ^177^Lu-DOTATATE PRRT (7.4 GBq/cycle), to which one showed partial response and two showed stable disease. One patient was previously treated with ^177^Lu-DOTA-EB-TATE (1.11 GBq/cycle) and demonstrated progressive disease. After repeated PRRT with 3.7 GBq/cycle of ^177^Lu-DOTA-EB-TATE, 3 patients (3/4) showed partial response and the remaining one patient (1/4) showed stable disease on ^68^Ga-DOTATATE PET/CT. An example is shown in Figure 2.

### Progression-free survival and overall survival

Follow-up data was available and ranges from 5 to 57 months after the start of the first PRRT in this study. The median follow-up period was 46 months (interquartile range, 13-50 months). The median PFS was 36 months, while the median OS was not reached. The observed PFS rates at 12 months, 24 months, and 36 months were 79.5%, 60.7%, and 47.2%, respectively; the observed OS rates at 12 months, 24 months, and 36 months were 92.9%, 79.7%, and 59.8%, respectively. In the univariate analysis, Ki-67 index was the only variable found to be significantly associated with PFS (Table 5). Therefore, a multivariate analysis was not performed. Of 14 patients with stable disease by RECIST criteria, those with partial response by PERCIST had longer PFS (median: not reached vs. 33 months; *P* = 0.817) and OS (median: not reached vs. 35 months; *P* = 0.109) than those with no response, whereas the difference was not statistically significant, probably due to the limited number of patients. Kaplan-Meier curves for PFS and OS after PRRT in this study are shown in Supplemental Figures 2 and 3.

## Discussion

Hematological and renal toxicity are considered as the main side effects and dose-limiting factors for PRRT. In this study, the rate of severe hematotoxicity rate was rather low with 13.3% of the patients experiencing grade 3 thrombocytopenia, and 3.3% experiencing grade 3 anemia, who presented with grade 3 anemia already at baseline. This rate was similar to the previously reported ^177^Lu-DOTATATE PRRT studies that described grade 3/4 hematotoxicity rate of 3.1%-15% 7, 18-24. Recently, Garske-Román *et al.* reported data on 200 NET patients receiving ^177^Lu-DOTATATE, and grade 3/4 hematotoxicity was found in 30 patients (15%) 25. In the study of Bergsma *et al.*, grade 3/4 hematotoxicity was observed in 11% of patients treated with ^177^Lu-DOTATATE PRRT 19. For the 4 patients presented with grade 3 thrombocytopenia in this study, all were previously treated with multiple courses of chemotherapy or sulfatinib/everolimus, which are risk factors associated with hematotoxicity 26. In addition, the platelet counts at baseline in 2 of them were only slightly higher than the lower limits (109 and 118 × 10^9^/L; normal 100-300 × 10^9^/L). These data confirmed the importance of baseline myeloid function and careful evaluation of risk factors for relevant toxicity. Regarding nephrotoxicity, no grade-2/3/4 toxicity was observed in our cohort, and renal function demonstrated by creatinine remained stable during the therapy.

Long-term hematotoxicity and nephrotoxicity are of particular concern in this trial. On follow-up, we observed low incidence of grade 3 hematotoxicity (3.4%) and no nephrotoxicity of any grade. Acute leukemia and MDS are rare delayed side effects and severe complications related to PRRT, which have been reported previously to occur in approximately 2% of patients 22. In our study, none of the patients developed MDS or leukemia during the follow-up period. Our data confirm that PRRT with ^177^Lu-DOTA-EB-TATE is a relatively safe therapeutic procedure in view of the low occurrences of short- and long-term toxicity.

In terms of efficacy, we observed a response rate of 33.3% and a disease control rate of 85.2% on RECIST criteria, and a response rate of 48.3% and a disease control rate of 86.2% on PERCIST criteria. These results are not inferior to that reported for ^177^Lu-DOTATATE PRRT. Recently, Sitani *et al.* retrospectively analyzed 468 NET patients who underwent PRRT with ^177^Lu-DOTATATE (dose: 5.55 to 7.4 GBq/cycle). They found a response rate of 30% and 31%, and a disease control rate of 90% and 88% on RECIST and PERCIST criteria, respectively 27. Another study performed by Parghane *et al.* analyzed 57 GEP-NET patients receiving PRRT with ^177^Lu-DOTATATE (dose: 7.4 GBq/cycle). They reported a response rate of 40% and 31%, and a disease control rate of 92.9% and 94.7% referring to RECIST and PERCIST criteria, respectively 28. In a recent meta-analysis on the efficacy of ^177^Lu-DOTATATE in NET patients, the pooled response rate and disease control rate were 27.58% and 79.14%, respectively, based on the RECIST criteria 29. These findings suggested that 3.7 GBq/cycle of ^177^Lu-DOTA-EB-TATE seems to be as effective as 5.55-7.4 GBq of ^177^Lu-DOTATATE for tumor control.

Treatment options are limited for patients who experience recurrence after PRRT, which led to the investigation of potential practice of PRRT re-treatment. Several studies have reported a safety profile for PRRT re-treatment that is similar to initial PRRT 30-32. However, the performance of PRRT re-treatment was reported to be poorer than initial PRRT 32, 33. The relatively poor performance of PRRT re-treatment was probably because patients have acquired a level of radio-resistance after previous PRRT. In the present study, 4 patients in our cohort were previously treated with PRRT, and repeated PRRT with ^177^Lu-DOTA-EB-TATE performed well for tumor control. Our preliminary findings indicate the potential benefit of ^177^Lu-DOTA-EB-TATE in PRRT re-treatment setting. In the future, it would make sense to apply ^177^Lu-DOTA-EB-TATE to PRRT re-treatment patients to investigate its potential value in this special setting.

In the present study, we observed a median PFS of 36 months after a median follow-up of 46 months, while the median OS was not reached. Our result compares favorably with those of previous ^177^Lu-DOTATATE studies, which reported a median PFS ranging from 26 to 37 months 4, 18, 23, 34. Ezzidin *et al*. in a retrospective study of 74 patients with metastatic NETs treated with ^177^Lu-DOTATATE, reported a median PFS of 26 months 35. In a large group of 443 NET patients treated with ^177^Lu-DOTATATE, Brabander *et al*. reported a median PFS of 29 months 5. In a recent study, Kennedy *et al*. retrospectively reviewed the long-term survival of 104 patients with advanced NETs treated with ^177^Lu-DOTATATE and reported a median PFS of 37 months 36. In the absence of a randomized trial comparing ^177^Lu-DOTA-EB-TATE with ^177^Lu-DOTATATE, these data seem to indicate that the survival benefit obtained with ^177^Lu-DOTA-EB-TATE at lower dose is not inferior to ^177^Lu-DOTATATE, at least in terms of PFS. We also found that Ki-67 index of greater than 10% was significantly associated with poorer PFS. A previous study has also reported that higher Ki-67 index was associated with poorer prognosis in NET patients treated with ^177^Lu-DOTATATE PRRT 35, and this finding warrants further investigation.

This study has several limitations. First, the main limitation of this study is the small population size, which might lead to some bias. The number of PRRT re-treatment patients was also limited. Still, our preliminary data were the first to report the potential benefit of ^177^Lu-DOTA-EB-TATE in this setting, encouraging study with larger sample size to provide stronger evidence for the effect of ^177^Lu-DOTA-EB-TATE in PRRT re-treatment patients. The second limitation is the lack of control group of standard PRRT with ^177^Lu-DOTATATE. Thus, the performance of ^177^Lu-DOTA-EB-TATE in this study was compared with the published literature. In the future, we will design a randomized controlled trial to compare ^177^Lu-DOTA-EB-TATE and standard ^177^Lu-DOTATATE to further determine the value of ^177^Lu-DOTA-EB-TATE. The third limitation is the limited length of follow-up period. Despite this shortcoming, we were able to report the long-term toxicity and survival outcome of ^177^Lu-DOTA-EB-TATE for the first time. Furthermore, the fact that most patients did not complete the planned 3 cycles due to various reasons may have affected the reported toxicity profile of ^177^Lu-DOTA-EB-TATE in this study.

## Conclusion

Our results suggest that PRRT with approximately 3.7 GBq ^177^Lu-DOTA-EB-TATE has acceptable toxicity profile and is effective in treating metastatic NET with high disease control rate. In addition, ^177^Lu-DOTA-EB-TATE achieved a favorable survival outcome with encouraging PFS. Future prospective randomized studies in larger number of NET patients comparing ^177^Lu-DOTA-EB-TATE with standard ^177^Lu-DOTATATE are warranted.

## Supplementary Material

Supplementary figures and table.

## Figures and Tables

**Table 1:** Demographic and baseline clinical characteristics of patients

Characteristic	Number	Percentage (%)
Male	17	56.7
Female	13	43.3
Age (years)	50.1 ± 11.8	
Primary tumor site		
Pancreas	18	60
Rectum	5	16.7
Small intestine	4	13.3
Paraganglioma	2	6.7
Cancer of unknown primary	1	3.3
Tumor grade		
G1	4	13.3
G2	24	80
G3	2	6.7
Treatment before PRRT		
Surgery	17	56.7
Somatostatin analogue	20	66.7
Chemotherapy	12	40
Everolimus	4	13.3
Radiotherapy	1	3.3
Tyrosine kinase inhibitor	9	30
Previously treated with PRRT	4	13.3
Transarterial chemoembolization	4	13.3
Metastases		
Liver	28	93.3
Bone	9	30
Lymph nodes	11	36.7
Lung	4	13.3

**Table 2:** Treatment cycles and cumulative administered activity for^ 177^Lu-DOTA-EB-TATE

Number of PRRT cycles	Number of patients (n = 30)	Percentage (%)	Cumulative activity (GBq)
1	4	13.3%	3.97 ± 0.24
2	13	43.3%	7.78 ± 1.07
3	13	43.3%	11.36 ± 2.01

**Table 3:** Hematotoxicity, hepatotoxicity, and nephrotoxicity before and after therapy according to CTCAE 5.0

Toxicity	CTC-grade	Baseline	1^st^ cycle		2^nd^ cycle		3^rd^ cycle	
			2 weeks	4 weeks	2 weeks	4 weeks	2 weeks	4 weeks
Leukopenia	Grade-1	4	3	2	3	6	3	2
	Grade-2	0	3	3	3	4	3	2
	Grade-3	0	0	0	0	0	0	0
	Grade-4	0	0	0	0	0	0	0
Thrombocytopenia	Grade-1	0	3	3	1	3	1	3
	Grade-2	0	0	0	1	1	1	0
	Grade-3	0	0	2	1	1	1	0
	Grade-4	0	0	0	0	0	0	0
Anemia	Grade-1	1	6	2	4	4	4	3
	Grade-2	2	0	2	1	1	0	1
	Grade-3	1	0	1	0	0	0	0
	Grade-4	0	0	0	0	0	0	0
Nephrotoxicity	Grade-1	6	1	2	1	1	1	0
	Grade-2	1	0	0	0	0	0	0
	Grade-3	0	0	0	0	0	0	0
	Grade-4	0	0	0	0	0	0	0
Hepatoxicity	Grade-1	3	1	3	2	1	1	0
	Grade-2	2	0	0	0	0	0	0
	Grade-3	0	0	1	0	0	0	0
	Grade-4	0	0	0	0	0	0	0

**Table 4:** Long-term hematotoxicity, nephrotoxicity, and hepatotoxicity according to CTCAE 5.0

Toxicity	Grade 1 (no. of patients)	Grade 2 (no. of patients)	Grade 3 (no. of patients)	Grade 4 (no. of patients)
Leukopenia	1	3	0	0
Thrombocytopenia	1	0	1	0
Anemia	1	2	0	0
Nephrotoxicity	0	0	0	0
Hepatoxicity	1	0	0	0

**Table 5:** Univariate Cox proportional hazards regression analysis of progression-free survival (PFS) and overall survival (OS)

Factor	PFS			OS		
	HR	95%CI	*P*	HR	95%CI	*P*
Gender						
Female	1			1		
Male	1.083	0.330-3.554	0.896	2.612	0.504-13.543	0.253
Age						
≤ 55 y	1			1		
> 55 y	1.318	0.401-4.330	0.649	2.188	0.488-9.806	0.306
Primary tumor type						
Non-pancreatic	1			1		
Pancreatic	0.523	0.159-1.721	0.286	0.308	0.060-1.594	0.160
Ki-67 index						
≤ 10%				1		
> 10%	4.973	1.430-17.293	0.012	1.527	0.341-6.838	0.580
Number of organs involved						
≤ 1	1			1		
> 1	0.309	0.082-1.169	0.084	0.898	0.200-4.035	0.888
Bone metastases						
No	1			1		
Yes	2.135	0.623-7.313	0.227	1.260	0.242-6.552	0.783
Hepatic tumor burden						
≤ 50%	1			1		
> 50%	1.183	0.360-3.892	0.782	1.680	0.375-7.524	0.498
Surgery of primary tumor						
No	1			1		
Yes	1.773	0.513-6.120	0.365	2.335	0.447-12.199	0.315
Chemotherapy						
No	1			1		
Yes	2.849	0.826-9.817	0.097	1.140	0.255-5.100	0.864

## Abbreviations

PRRT: peptide receptor radionuclide therapy
NETs: neuroendocrine tumors
CTCAE: Common Terminology Criteria for Adverse Events
PFS: progression-free survival
OS: overall survival
SSTRs: somatostatin receptors
EMA: European Medicines Agency
FDA: Food and Drug Administration
EB: Evans Blue
