# Supplementary material for: Safety and efficacy of peptide receptor radionuclide therapy with 177Lu-DOTA-EB-TATE in patients with metastatic neuroendocrine tumors
Source: Theranostics. 2022 Sep 6;12(15):6437–45. doi: 10.7150/thno.77219 (PMC9516233; doi:10.7150/thno.77219)
Supplement: Supplementary file 1 — Supplementary figures and table. [file thnov12p6437s1.pdf]

Table S1. Demographic and baseline clinical characteristics of patients

Patient	Age (years)	Gender	Primary site	Grade (Ki-67 index)	Previous treatment	Number of PRRT cycles
**1**	66	M	Paraganglioma	G2 (5%)	SSA, surgery, TACE, PRRT	3
**2**	39	F	Duodenum	G2 (5%)	SSA	2
**3**	55	M	Rectum	G2 (15%)	Surgery, SSA	2
**4**	15	F	Pancreas	G2 (5%)	Surgery, SSA	1
**5**	49	M	Pancreas	G2 (5%)	SSA, chemotherapy	3
**6**	41	F	Pancreas	G2 (15%)	Surgery, SSA	3
**7**	34	M	Pancreas	G2 (10%)	Surgery, SSA, chemotherapy, everolimus, tyrosine kinase inhibitor	3
**8**	39	M	Pancreas	G2 (3%)	SSA, TACE, PRRT	2
**9**	60	M	Rectum	G2 (9%)	SSA	2
**10**	57	F	Pancreas	G2 (5%)	Surgery, SSA, everolimus	2
**11**	43	M	Pancreas	G2 (10%)	SSA, chemotherapy, tyrosine kinase inhibitor	2
**12**	60	M	Pancreas	G2 (3%)	SSA, chemotherapy, tyrosine kinase inhibitor	2
**13**	55	F	Pancreas	G2 (20%)	Surgery, SSA, PRRT	2
**14**	51	F	Pancreas	G2 (5%)	Surgery, SSA, PRRT	1
**15**	64	M	Rectum	G2 (8%)	Surgery, SSA, everolimus	3
**16**	69	M	Pancreas	G3 (30%)	Surgery, chemotherapy, tyrosine kinase inhibitor	1
**17**	45	M	Pancreas	G2 (15%)	Surgery, radiotherapy, TACE, SSA, chemotherapy	3
**18**	48	M	Duodenum	G1 (1%)	Surgery, tyrosine kinase inhibitor,	3
**19**	56	F	Pancreas	G3 (25%)	Chemotherapy	3
**20**	59	M	Rectum	G2 (15%)	Surgery, chemotherapy	2
**21**	46	F	Duodenum	G2 (18%)	Surgery, SSA	3
**22**	34	M	Duodenum	G2 (10%)	TACE, chemotherapy	3
**23**	63	M	Pancreas	G2 (5%)	Tyrosine kinase inhibitor	1
**24**	58	F	Pancreas	G2 (3%)	Surgery, SSA	2
**25**	42	F	Pancreas	G2 (5%)	Tyrosine kinase inhibitor	3
**26**	66	M	Rectum	G2 (5%)	Tyrosine kinase inhibitor, everolimus	2
**27**	42	F	Pancreas	G1 (<2%)	Surgery, SSA, tyrosine kinase inhibitor, chemotherapy	2
**28**	45	M	Pancreas	G1 (1%)	Surgery	3
**29**	56	F	Paraganglioma	G2 (15%)	Chemotherapy	3
**30**	48	F	Cancer of unknown primary	G1 (2%)	SSA	2

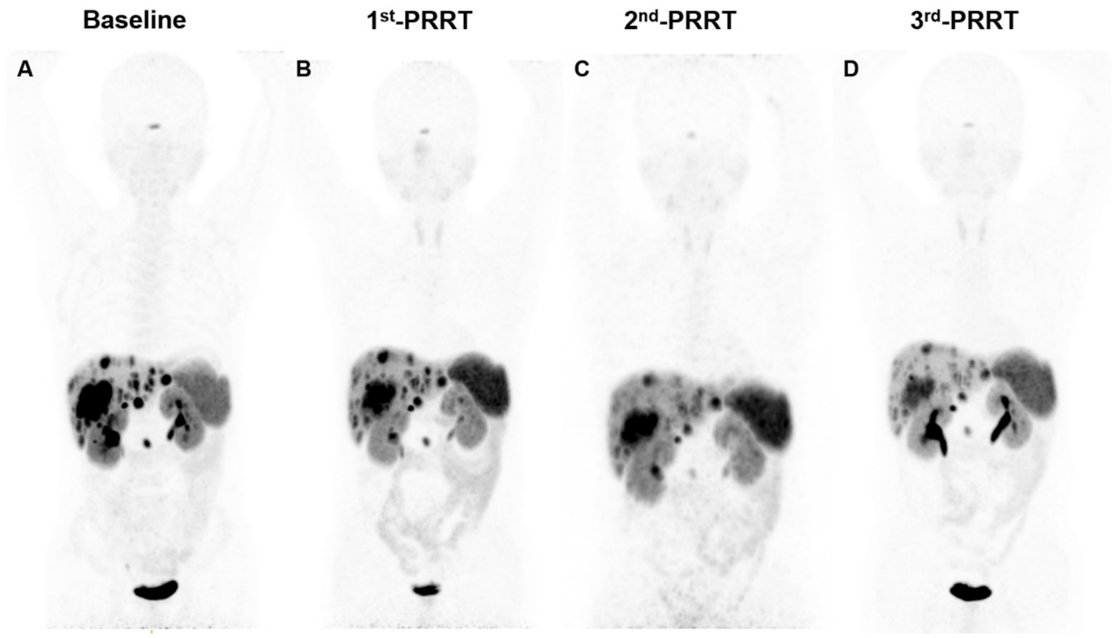

Figure S1. ^{68}Ga-DOTATATE PET/CT images of a representative patient with metastatic pancreatic NET for response evaluation after each cycle of ^{177}Lu-DOTA-EB-TATE (**A**: baseline ^{68}Ga-DOTATATE PET/CT image; **B**: restaging after 1 cycle; **C**: restaging after 2 cycles; **D**: restaging after 3 cycles).

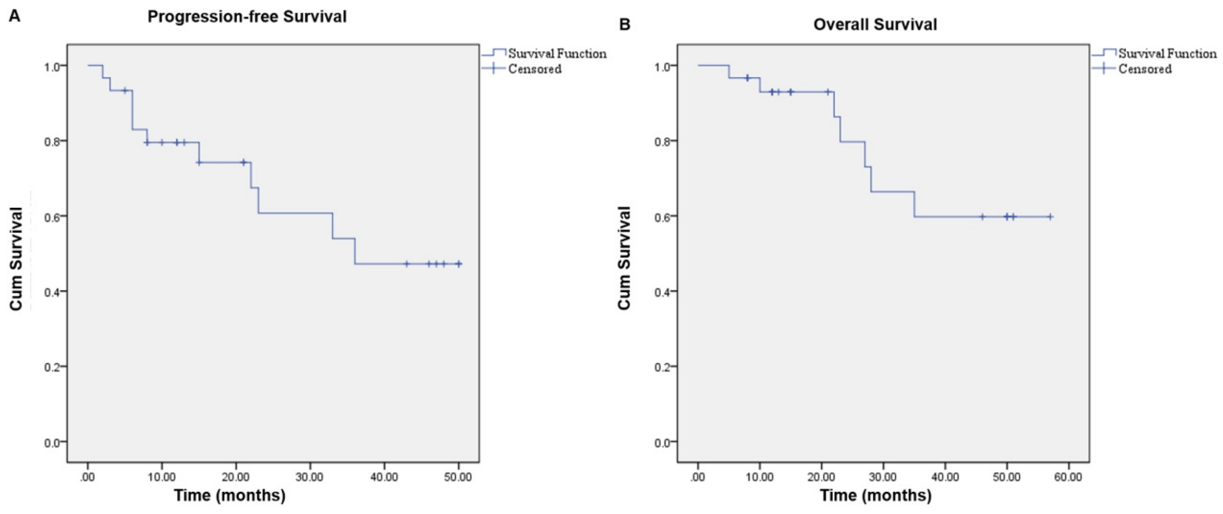

Figure S2. Kaplan-Meier curves for progression-free survival (**A**) and overall survival (**B**).

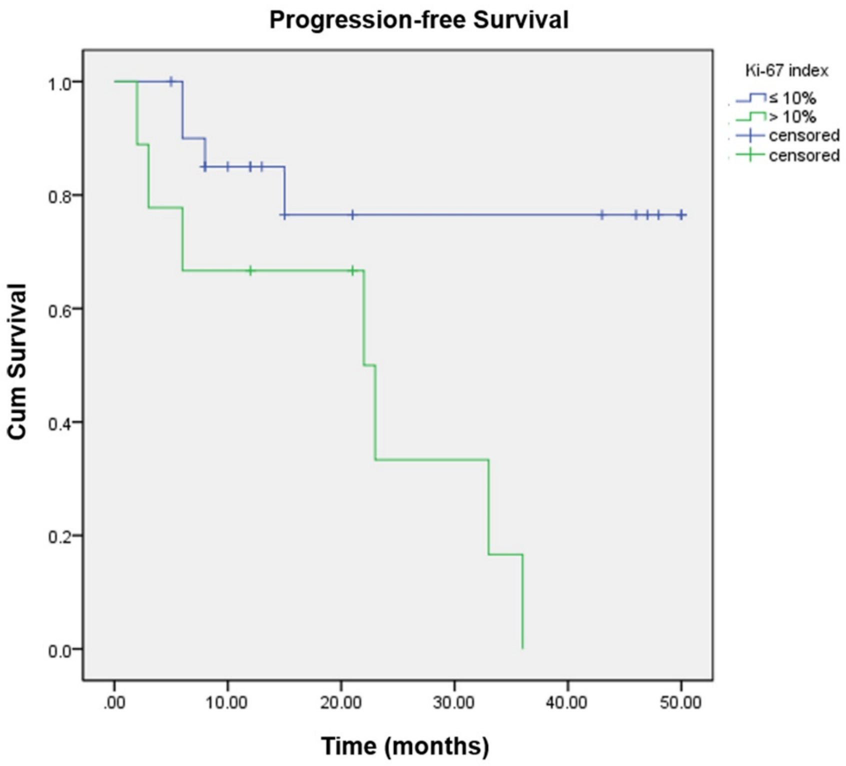

Figure S3. Kaplan-Meier curve for progression-free survival stratified by tumor proliferation index (Ki-67 index of ≤ 10% vs. > 10%).
